# Important roles of *Hif1a* in maternal or adult BPA exposure induced pancreatic injuries

**DOI:** 10.1038/s41598-023-38614-8

**Published:** 2023-07-17

**Authors:** Huiping Liu, Yongnian Zhou, Yike Li, Zhihua Gong

**Affiliations:** 1 Department of Cardiopulmonary Function Examination, Shanxi Province Cancer Hospital/Shanxi Hospital Affiliated to Cancer Hospital, Chinese Academy of Medical Sciences/Cancer Hospital Affiliated to Shanxi Medical University, Taiyuan, 030013 China; 2 Department of Clinical Laboratory, Shanxi Bethune Hospital, Shanxi Academy of Medical Sciences, Tongji Shanxi Hospital, Third Hospital of Shanxi Medical University, Taiyuan, 030032 China; 3 Tongji Hospital, Tongji Medical College, Huazhong University of Science and Technology, Wuhan, 430030 China; 4 College of Environment and Resource, Research Center of Environment and Health, Shanxi University, Taiyuan, 030006 China

**Keywords:** Computational biology and bioinformatics, Environmental social sciences, Diseases

## Abstract

Bisphenol A (BPA) is a monomer to produce polycarbonate plastics and can be released into the environment through human activities, leading to its accumulation in animals, plants and humans through direct contact or environmental exposure. Epidemiological studies have reported that BPA exposure is associated with metabolic disorders. The pancreas is an important endocrine organ and plays an important role in metabolic disorders. To explore the possible long-term effects of BPA exposure on neonatal health, bioinformatic methods were used to identify differentially expressed genes (DEGs) by comparing the neonatal pancreas after maternal exposure to BPA with the adult pancreas after direct exposure to BPA. Two datasets about BPA exposure and pancreatic abnormality, GSE82175 and GSE126297 in Gene Expression Omnibus (GEO) at the National Center for Biotechnology Information (NCBI) were collected. Control (or BPA-exposed) offspring (maternal exposure) and Control (or BPA-exposed) adults (direct exposure) were defined as Control (or BPA) groups. The results showed that BPA disturbed the normal function of the pancreas in both offspring and adults, with offspring showing higher susceptibility to BPA than adults. Seventeen insulin secretion-related DEGs (*Stxbp5l*, *Fam3d*, *Mia3*, *Igf1*, *Hif1a*, *Aqp1*, *Kif5b*, *Tiam1*, *Map4k4*, *Cyp51*, *Pde1c*, *Rab3c*, *Arntl*, *Clock*, *Edn3*, *Kcnb1*, *and Krt20*) in the BPA group were identified, and 15 regulator DEGs (*Zfp830*, *4931431B13Rik*, *Egr1*, *Ddit4l*, *Cep55*, *G530011O06Rik*, *Hspa1b*, *Hspa1a*, *Cox6a2*, *Ibtk*, *Banf1*, *Slc35b2*, *Golt1b*, *Lrp8*, *and Pttg1*) with opposite expression trends and a regulator gene *Cerkl* with the similar expression trend in the Control and BPA groups were identified. *Hif1α* might be an important molecular target for pancreatic cancer caused by BPA exposure, and pregnancy is a critical window of susceptibility to BPA exposure.

## Introduction

Bisphenol A (BPA) is a monomer to produce polycarbonate plastics, which were mainly used for plastic bags, water and milk bottles, food and drink cans, thermal paper, dental materials, and medical devices^1–3^. As a constituent of food containers and packaging, BPA can leach into food products, especially after heating^4^. For most people, the primary source of exposure to BPA is through the digestive system, which accounts for more than 90% of total BPA^3^. Furthermore, vertical transmission and the respiratory and cutaneous systems are also possible sources of exposure^4^. Calafat et al. reported that BPA was detected in the urine of children and adults ranging from 0.4 to 149 ng/ml^5^. Multiple studies have reported that BPA can be detected in maternal serum (< 0.2–153.5 ng/ml)^6,7^, umbilical cord serum (< 0.05–52.26 ng/ml)^8^ and breast milk (≤ 0.22–10.8 ng/ml)^9^. In particular, the existence of free BPA in maternal and fetal serum and breast milk may result in long-term exposure to BPA during the fetal and neonatal periods, thereby exerting harmful effects on fetuses and neonates.

BPA is one of the well-known endocrine-disrupting chemicals (EDCs), which affect the synthesis, metabolism, and function of hormones. In 1936, Dowds and Lawson first discovered the estrogenic properties of BPA^10^, which can bind and activate classical estrogen receptors (ERα and ERβ) to affect both body weight and tumorigenesis^4^. Growing evidence supports that BPA has an anti-androgen effect. After entering the body, BPA can compete with 5α-dihydrotestosterone (DHT) to bind to androgen receptors (ARs), thereby disrupting the normal function of androgens and causing the dysfunction of reproduction and development^11^. Epidemiologic studies have also reported BPA exposure is associated with metabolic disorders, such as obesity and type-2 diabetes (T2DM)^12^. Animal studies manifest that BPA can either directly act on pancreatic cells and impair insulin and glucagon secretion, or act on adipose cells, muscle and hepatic function to trigger an insulin-resistant state^13^. Moreover, increasing evidence indicates that low levels of BPA (40 μg/kg/day or 50 μg/kg/day) exposure during pregnancy and the perinatal period can affect glucose tolerance in offspring and mothers through epigenetic alterations or maternal metabolic dysfunction^14–16^. The hypothesis of fetal origins of adult diseases (FOAD) suggests that environment-related fetal stress can enhance the risk of adult diseases^17^. Garcia-Arévalo et al. found that exposure to BPA during pregnancy affected β-cell mass in mouse offspring by initiating estrogen-signaling mechanisms in fetuses, which may contribute to impaired glucose tolerance during adulthood^18^. More and more animal studies manifest that exposure to endocrine disruptors during gestation could negatively impact fetal and placental health by interfering with the embryonic developing epigenome, thus establishing disease paths into adulthood.

Pancreas is an accessory organ located in the abdomen and surrounded by the stomach, liver, small intestine, and other organs. It produces exocrine enzymes to help with digestion and endocrine hormones to help regulate blood glucose^19^. Acute pancreatitis, chronic pancreatitis, cystic fibrosis, pancreatic cancer, and diabetes mellitus are common pancreatic diseases^20^. Global estimates of incidences of acute pancreatitis, chronic pancreatitis, and pancreatic cancer are about 33.74, 9.62, and 8.14 cases per 100,000 person-years, respectively^21^. Acute pancreatitis is a common acute gastrointestinal disease characterized by a local and systemic inflammatory response^22^. The long-term effects of acute pancreatitis are considerable, with patients developing recurrent episodes of acute pancreatitis, progressing to chronic pancreatitis, and developing endocrine and exocrine insufficiency^23^. Chronic pancreatitis is defined as a pathological fibro-inflammatory with irreversible damage to the pancreas, and can be induced by toxic factors, metabolic abnormalities, obstructive mechanisms, genetics, and autoimmune responses^24^.

In order to explore the possible effects of maternal factors on the risk of pancreatic cancer in offspring after maternal or direct exposure to BPA, bioinformatic methods were used to identify differentially expressed genes (DEGs) by comparing gene expression profiles of the pancreas between offspring and adults. Furthermore, possible key biomarkers were identified.

## Materials and methods

### Data collection for BPA exposure and pancreatic abnormality

Using the following key-words, *BPA*, *exposure*, and *pancreas*, 2 datasets (GSE82175 and GSE126297) were screened in Gene Expression Omnibus (GEO) at the National Center for Biotechnology Information (NCBI). GSE82175^25^ is related to maternal BPA exposure, and GSE126297^26^ is related to direct BPA exposure. Detailed exposure information can be seen in the original articles. Briefly, in GSE82175, pregnant mice were treated with vehicle or BPA (10 or 100 μg/kg/day) from day 9 to 16 of gestation. Pancreatic islets were isolated from male offspring at postnatal day 30 (P30). In GSE126297, a total of 100 μg/kg/day (two injections of 50 μg/kg/day) was administered in adult mice subcutaneously for four days, and pancreatic islets were collected sixteen hours after the last injection. The same volume of tocopherol-stripped corn oil (100 μL) was used as vehicle control. RNA was extracted and hybridized on Affymetrix microarrays (GPL1261, [Mouse430_2] Affymetrix Mouse Genome 430 2.0 Array). In order to ensure the consistency of exposure concentration, we selected the high-concentration exposure group in GSE82175 for follow-up analysis.

### Data processing procedure

In this study design, GSM2185622, GSM2185623, GSM2185626, and GSM2185627 collected in GSE82175, together with GSM3595790, GSM3595791, GSM3595792, GSM3595793, GSM3595794, and GSM3595795 collected in GSE126297, were imported into Rstudio (version: 2022.02.2-485) using R (version: 4.2.0). For each dataset, the gcrma and nsFilter functions of the GEOquery package (version: 2.64.0) were used for data normalization and filtration. After that, preprocessed data were merged using the dplyr package (version: 1.0.7) and batch effects of different samples were removed using the removeBatchEffect function in the limma package (version: 3.52.0). DEGs between Control (or BPA) in offspring (maternal exposure) and Control (or BPA) in adult (direct exposure) were screened with limma. Following this, DEGs between the two control groups and those between the two BPA groups were calculated, respectively. Finally, genes of the microarrays were annotated with mouse4302.db (version: 3.13.0) and annotate (version: 1.74.0) packages. Genes without annotation information were filtered. As for the genes with more than one probe, only the annotation that corresponded to the minimum probe ID was used for this gene in the following analysis.

### Gene set enrichment analysis (GSEA)

GSEA is a computational method that determines whether an a priori defined set of genes shows statistically significant, concordant differences between two biological states^27^. In the present study, the gseGO function of clusterProfiler (version: 4.4.1) was used to conduct GSEA for all annotated genes with their corresponding fold change (FC) in the Control group and BPA group, respectively. After that, the gseaplot and treeplot functions in the enrichplot package (version: 1.16.0) were used for data visualization of the top 10 and clustered top 30 terms of GSEA, respectively.

### Visualization of DEGs

Control (or BPA) in offspring (maternal exposure) and Control (or BPA) in adults (direct exposure) were defined as Control (or BPA) groups. DEGs in the Control and BPA groups were visualized by HeatMap and Volcano plot plug-in units of TBtools^28^. After importing the gene expression matrix of the Control and BPA groups, heatmaps were generated using the HeatMap plug-in. In addition, volcano plots were generated when DEGs with FC and *p*-values in the Control and BPA groups were imported. The intersection of DEGs between the Control and BPA groups was visualized using the Venn plug-in.

### Gene ontology (GO) analysis of DEGs

GO analysis of all DEGs in the Control and BPA groups, DEGs only in the Control or BPA group, and those in the intersection was conducted. Briefly, DEGs in symbol format were imported and converted to ENTREZID. The enrichGO function of clusterProfiler was used for GO enrichment, and the goplot and treeplot functions were used to visualize the top 10 and clustered top 30 terms of GO, respectively.

### Gene screening strategy

Based on the results of GO analysis, genes related to peptide regulation insulin secretion pathways, including *regulation of insulin secretion*, *regulation of protein secretion* and *peptide secretion*, were considered the key genes that participated in adverse effects induced by BPA exposure in the pancreas. All the genes in the intersection between DEGs of the Control and BPA groups were also considered regulators of the key genes.

### Gene interaction network performance

The lists of key genes and regulator genes were imported into the STRING database. The interaction between these genes was predicted. Different required interaction scores (0.150, 0.400, and 0.700) were set to get more interactive information. The results for the most interactive information were downloaded and imported into Cytoscape (version: 3.9.1). Moreover, genes with no interaction were also imported. Using the GeneMANIA plug-in, the network of the genes was generated.

### Disease prediction

Based on the above analysis, genes (nodes in the network) with no less than 5 interactively adjacent nodes were used for further disease analysis. Comparative Toxicogenomics Database (CTD) is a robust, publicly available database that aims to advance the understanding of how environmental exposures affect human health^29^. We used CTD to screen the mostly related disease for each gene. After that, we summarized the common ground of these diseases, i.e., whether they were related to one or more cancer symptoms, such as weight loss, carcinoma, neoplasms, inflammation, hyperplasia, metastasis, and fibrotic diseases. Then, the GEPIA database was used to predict gene expressions and disease-free survival curves for each gene in the tumor and normal population^30^.

## Results and discussion

It is well known that environmental chemical exposure can lead to adverse health effects on organ function. BPA, as a common environmental endocrine disruptor, has been confirmed to be related to disturbed endocrine function. For the datasets we referenced, the authors mainly focused on pancreatic β-cell growth in offspring (GSE82175) and pancreatic β-cell ion channels in adult (GSE126297). In GSE82175, changes in mRNA expression patterns in offspring were more prominent in islets in the BPA10 group, although a lower extent of changes was also observed in the BPA100 group. In addition, BPA exposure during pregnancy disrupted glucose homeostasis in mothers and adult male offspring^31^. More importantly, adverse effects induced by maternal environmental chemical exposure have attracted more researchers’ interest. However, most of the studies focused on maternal exposure itself but ignored potential differences between maternal and direct exposure under different exposure conditions. In another study, neonatal and adult female rats were injected subcutaneously with different doses of BPA (25 ng/kg/d or 5 mg/kg/d), the results show that neonatal exposure leads to effects occurring after exposure and persisting over the long term, while adult exposure causes transient effects during exposure^32^. This research emphasizes the important role of critical exposure windows. In the present study, molecular mechanisms underlying different effects of BPA on the pancreas between maternal and direct BPA exposure were explored.

### Effects of maternal and direct BPA exposure on the pancreas

**Figure 1:**
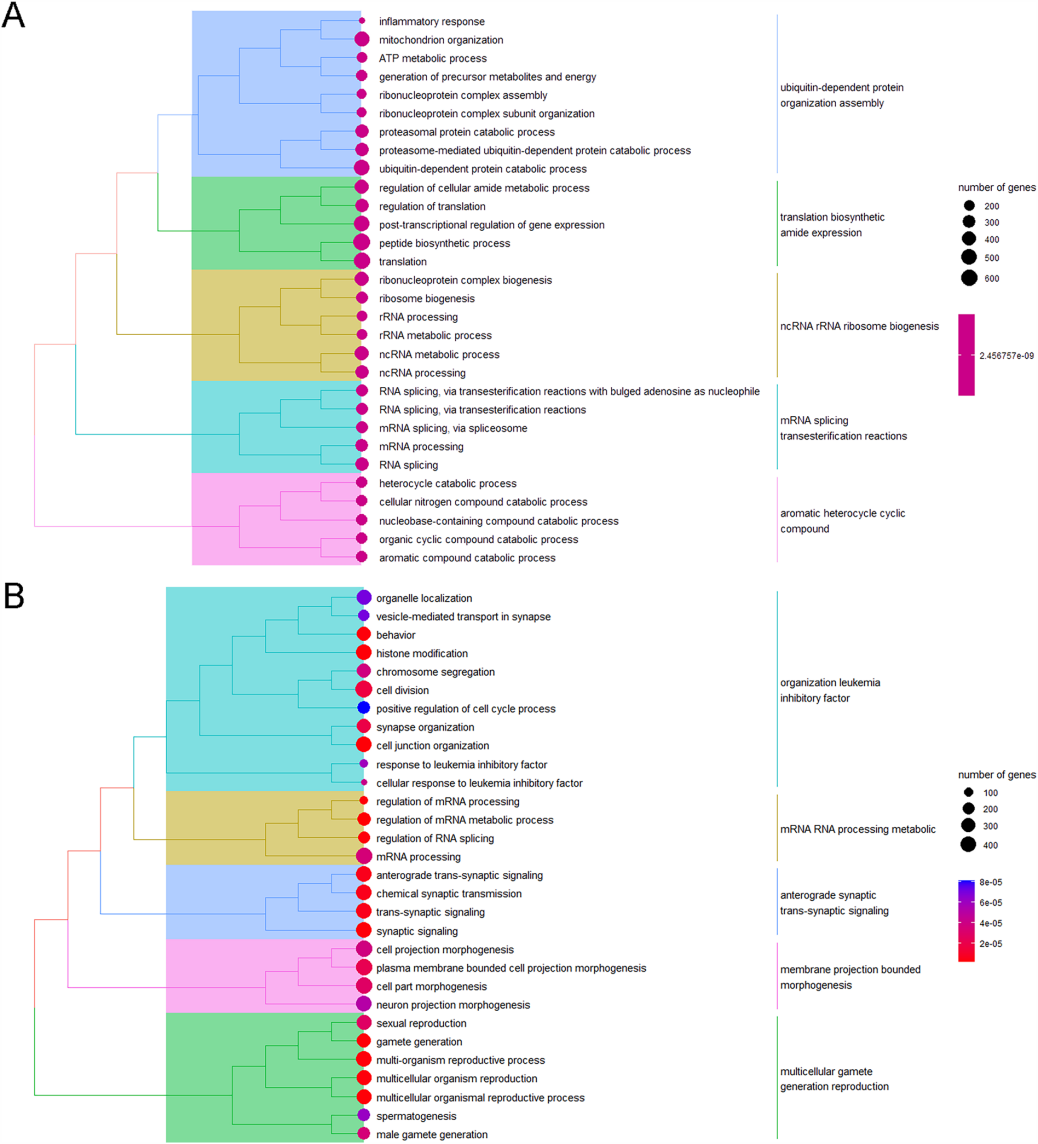
GSEA analysis for all genes with their corresponding FC values included in (**A**) the Control group and (**B**) the BPA group. FC value of a gene in the Control group (or in the BPA group) was calculated by dividing its average of control in offspring (maternal exposure research) by those in adults (direct exposure research). The size of the circle means the number of genes that participated in the certain GO term, numbers represent specific numerical values. The color of the circle means the significance (calculated adj-*p* value), i.e., the closer the color is close to blue, the lower the significance is, and the closer the color is close to red, the higher the significance is, numbers represent specific numerical values.

First, we reanalyzed the possibly activated biological pathways based on (1) GSEA of BPA exposure (GSEA in the BPA group, GSEA^BPA^, maternal vs. direct); (2) GSEA of vehicle exposure (GSEA in the Control group, GSEA^Con^, maternal vs. direct); (3) the comparison between GSEA^BPA^ and GSEA^Con^. The results showed that GSEA^Con^ were mainly enriched in *ubiquitin-depend protein organization assembly*, *translation biosynthetic amide expression*, *ncRNA rRNA ribosome biogenesis*, *mRNA splicing transesterification reactions*, and *aromatic heterocycle cyclic compound* (Fig. 1A). Those of GSEA^BPA^ were mainly biological processes related to *organization leukemia inhibitory factor*, *mRNA RNA process metabolic*, *anterograde synaptic trans-synaptic signaling*, *membrane projection bounded morphogenesis*, and *multicellular gamete generation reproduction* (Fig. 1B). Interestingly, both groups included the ribosome RNA-related metabolic processes or protein biosynthesis processes (Fig. S1), most of which were activated. This indicated that maternal exposure enhanced cell activities compared with those in direct exposure in both BPA and Control groups. However, detailed activated processes need to be further explored. It is known that BPA, as one of the EDCs, is closely associated with endocrine, immune, and oncological diseases. BPA can bind to hormone receptors, such as ERs, ARs and thyroid hormone receptors, and then directly regulate gene expression and protein biosynthesis. Shafei A et al. reported that BPA is not only associated with hormone-dependent cancers, such as breast cancer, ovarian cancer and prostate cancer, but also related to the increased risk of cervical cancer and lung cancer^33^.

### DEGs in the control and BPA groups when comparing maternal BPA exposure to direct exposure

**Figure 2:**
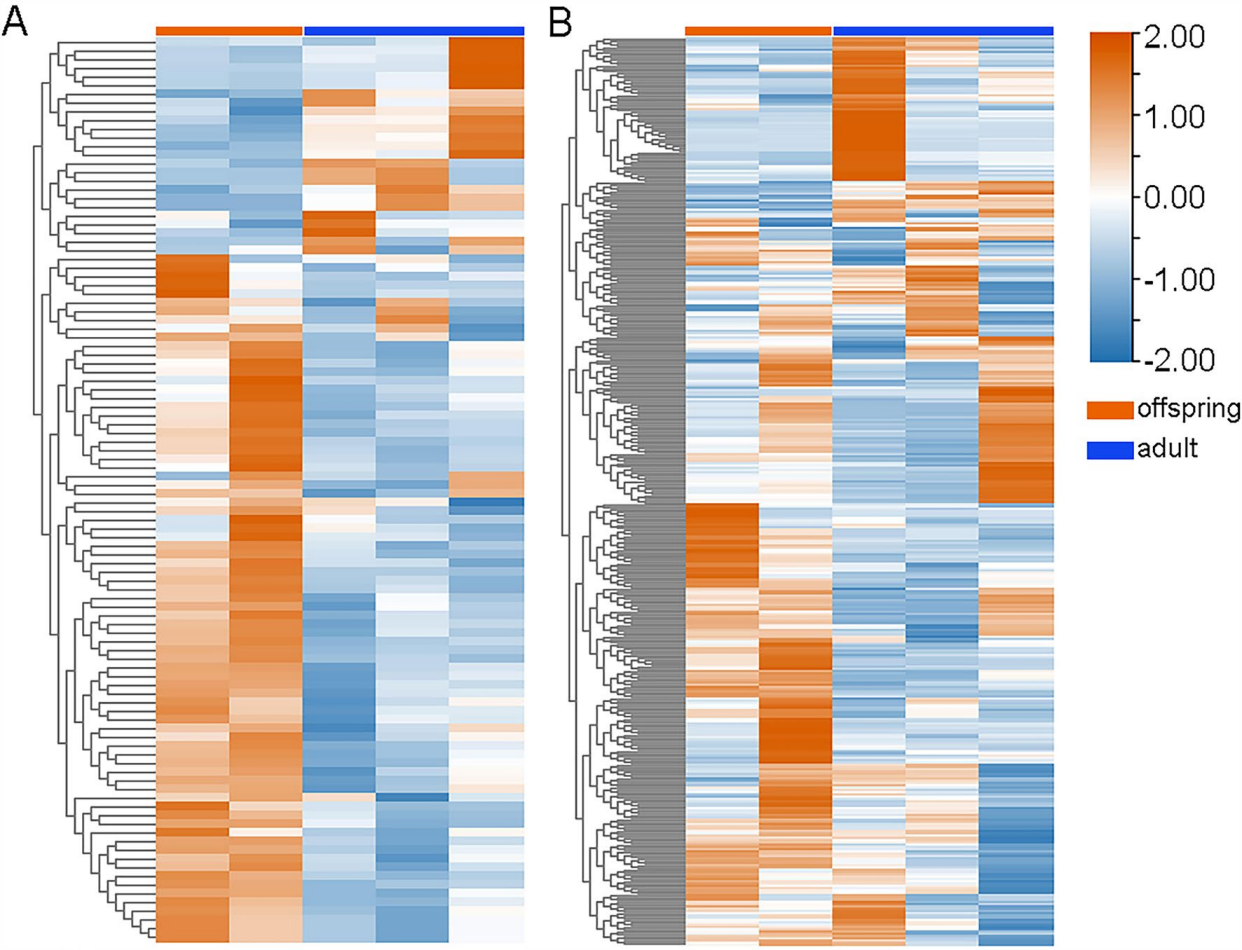
Heatmaps of DEGs in (**A**) the Control group and (**B**) the BPA group when comparing gene expressions of offspring (maternal exposure research) to those of adults (direct exposure research). Red or blue columns represent the genes in offspring or adults, respectively. Orange and blue mean up-regulated genes and down-regulated genes, respectively; the numbers represent the scales of the normalized expression values. Heatmaps were generated using the HeatMap plug-in in TBtools.

**Figure 3:**
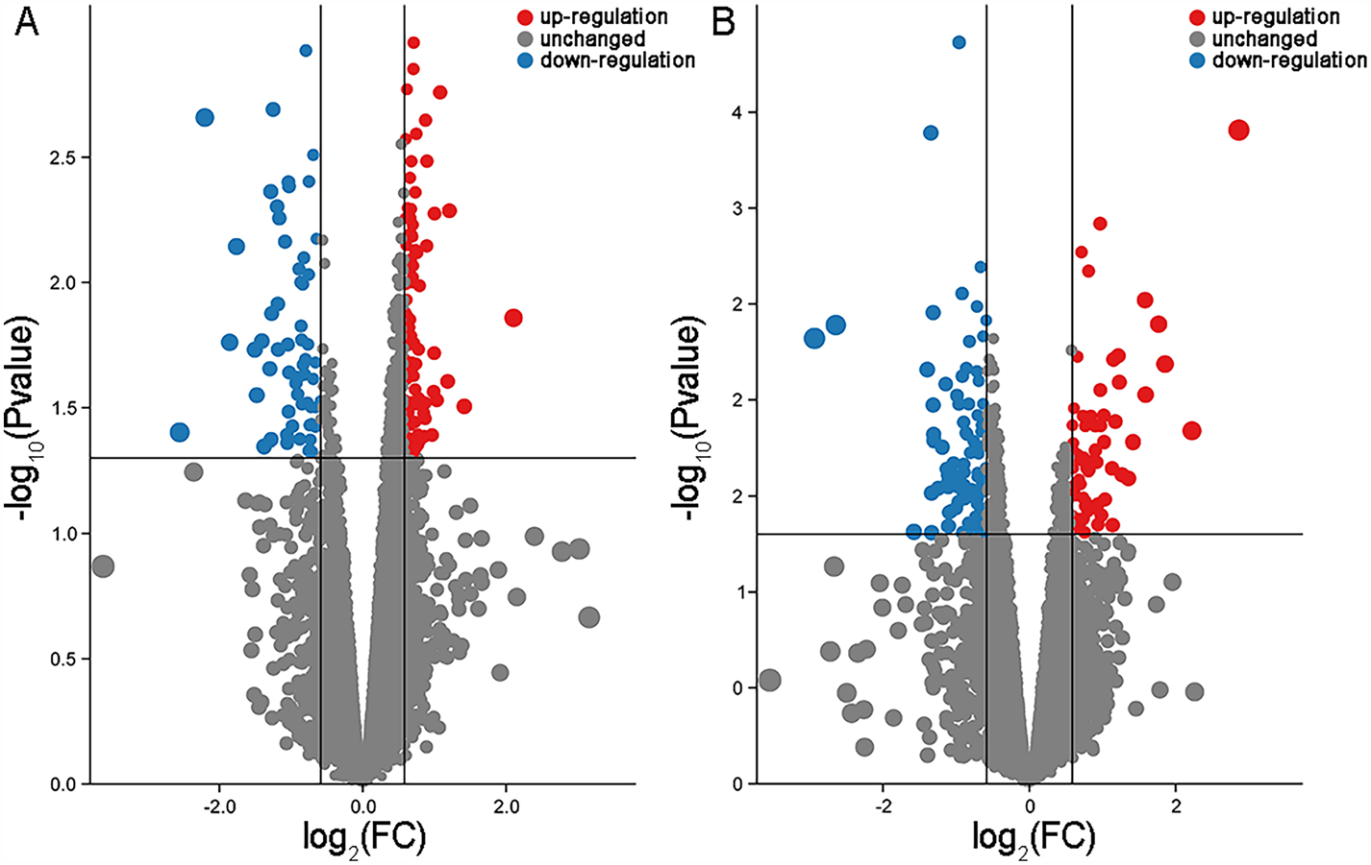
Volcano maps of DEGs in (**A**) the Control group and (**B**) the BPA group when comparing gene expressions of offspring (maternal exposure research) to those of adults (direct exposure research). Red or blue circles mean the genes were up-regulated or down-regulated. FC of each gene was scaled to the size of the circle.

**Figure 4:**
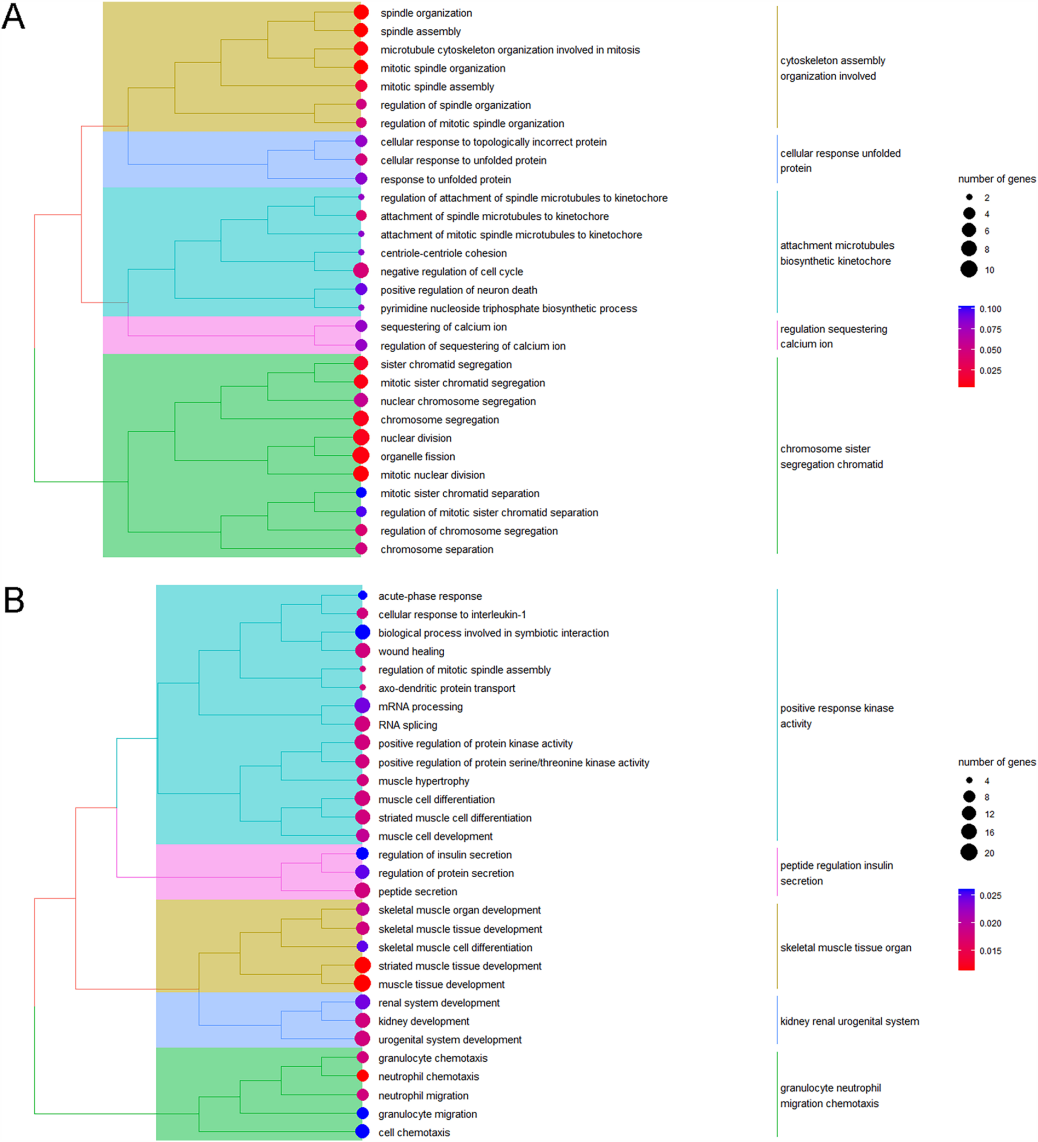
GO analysis for DEGs included in (**A**) the Control group and (**B**) the BPA group. The size of the circle means the number of genes that participated in the certain GO term, numbers represent specific numerical values. The color of the circle means the significance (calculated adj-*p* value), i.e., the closer the color is close to blue, the lower the significance is, and the closer the color is close to red, the higher the significance is, numbers represent specific numerical values.

Using GSEA, we could not distinguish the specially altered biological pathways in the Control and BPA groups when comparing maternal and direct exposure. As shown in Figs. 2 and 3, there were 99 (77 up-regulated and 22 down-regulated) and 354 (234 up-regulated and 120 down-regulated) DEGs in the Control and BPA groups, respectively. We next conducted GO enrichment for DEGs in different groups. As shown in Figs. 4A, S2, and Table S1, *cytoskeleton assembly-*, *protein*-, *kinetochore*-, *calcium ion*-, and *chromosome sister segregation*-related pathways in the Control group were more activated in offspring compared to adults. In the BPA group (Figs. 4B, S3, and Table S2), we found that the offspring were more sensitive in *response to kinase activity*, *skeletal muscle and kidney developmental processes*, and *neutrophil migration*. Importantly, *peptide response insulin secretion* was enriched, which strongly implied that BPA disturbed the normal function of the pancreas and offspring showed higher susceptibility to BPA than adults.

Alonso-Magdalena et al.^34^ reported that environmentally relevant doses of BPA can up-regulate pancreatic β-cell insulin content by ERα activation. Others indicated that BPA exposure modulated the expression and function of Na^+^ and K^+^ channels via ERβ in mouse pancreatic islets^26^. Chang et al.^35^ revealed that maternal exposure to BPA (10 μg/kg/d) decreased pancreatic β-cell mass at birth by reducing pancreatic and duodenal homeobox 1 (PDX1^+^) progenitors and altering the histone modifications of *Pdx1* during fetal development, which finally disrupted glucose homeostasis in adulthood. Some studies also show that BPA exposure can modulate gene expression by DNA methylation and the transgenerational effects can lead to long-term damage^14,15^. Consistent with our results, another study about transgenerational effects induced by BPA exposure showed that BPA exposure could lead to smaller islets, suppressed β-cell mass, and enhanced glucose-stimulated insulin secretion in F3 adults^36^. In addition, Dos Santos et al. found that various EDCs, including BPA, tributyltin (TBT), triphenylphosphate (TPP), perfluorooctanoic acid (PFOA), dichlorodiphenyldichloroethylene (DDE) and triclosan (TCS), could lead to β-cell apoptosis, alter β-cell viability, and disturb glucose-stimulated insulin secretion in vitro^37^.

### Screening of key DEGs

**Figure 5:**
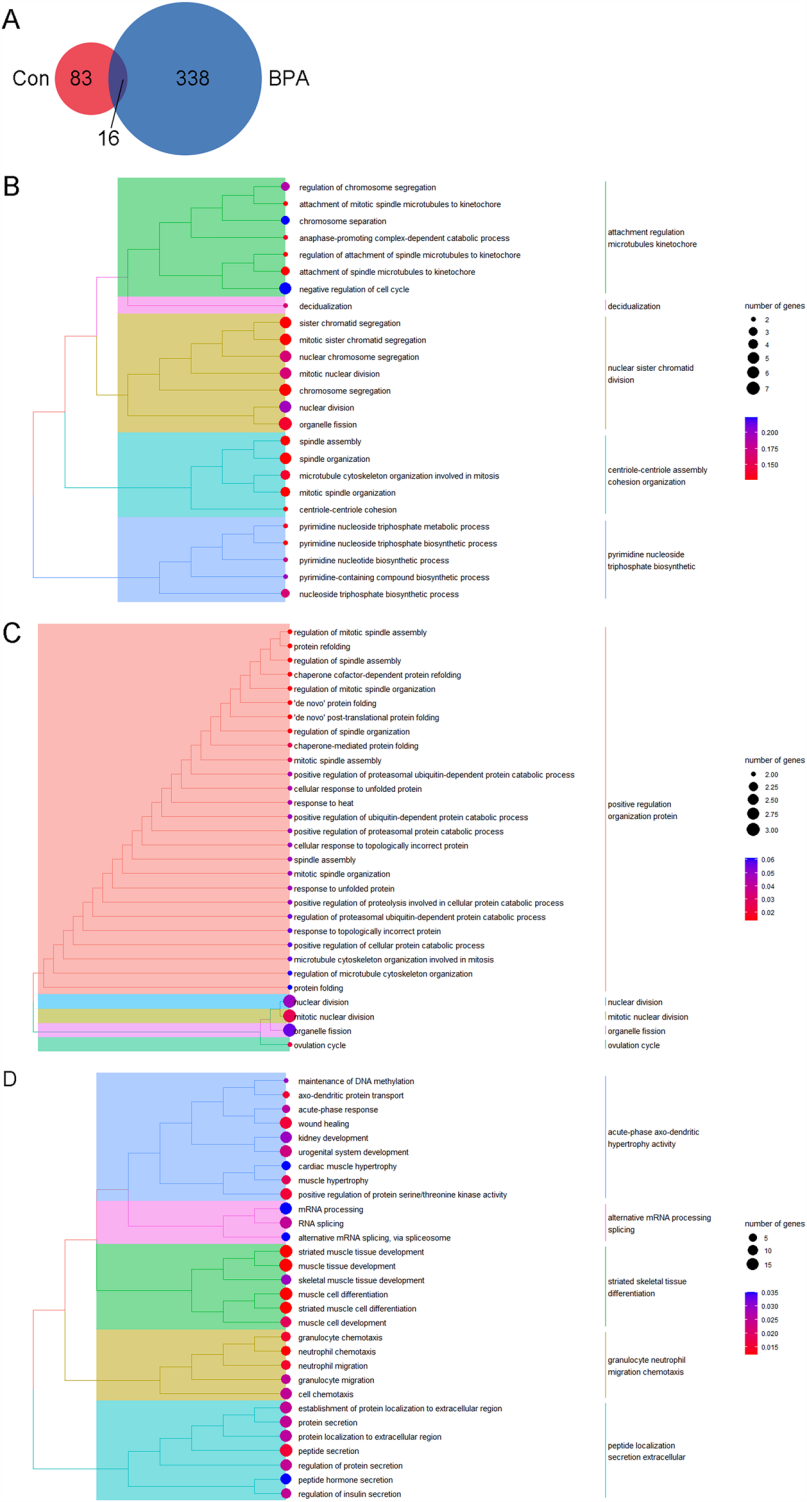
Comparison analysis for DEGs and the corresponding GO terms included in the Control group and BPA group. (**A**) Venn diagram of the DEGs in the Control and BPA groups, 83, 16, and 338 represent the DEGs only in the Control group, in both groups, and in the BPA group. GO terms enriched by DEGs (**B**) only in the Control group, (**C**) in both groups, and (**D**) in the BPA group. The size of the circle means the number of genes that participated in the certain GO term, numbers represent specific numerical values. The color of the circle means the significance (calculated adj-*p* value), i.e., the closer the color is close to blue, the lower the significance is, and the closer the color is close to red, the higher the significance is, numbers represent specific numerical values.

**Figure 6:**
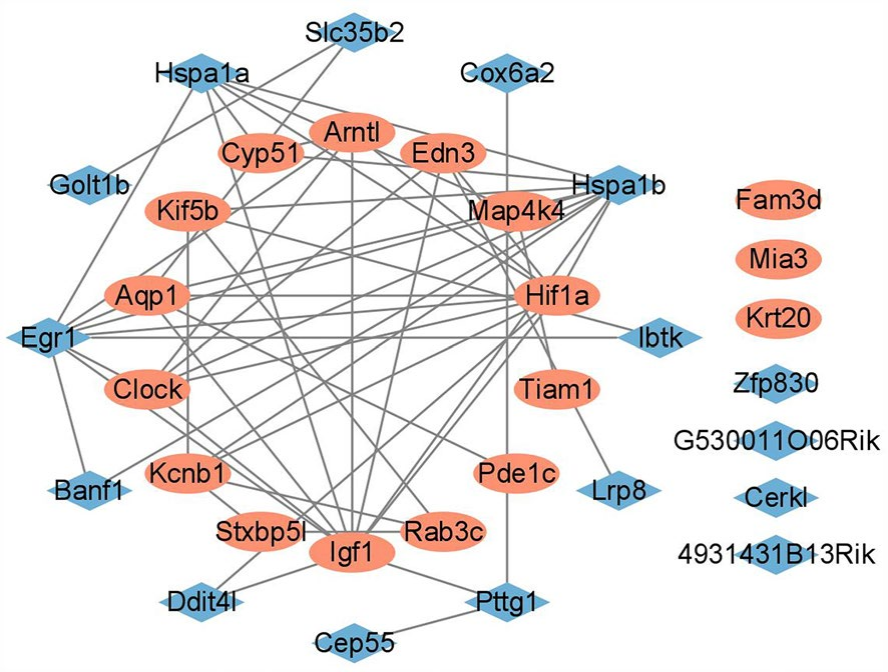
Gene interaction networks with the most interactive information generated by genes participated in peptide regulation insulin secretion-related pathways (key genes, shown in red) and the genes included in the intersection between the Control and BPA groups (regulatory genes, shown in blue). *Note* Fam3d and G530011O06Rik were recognized as Oit1 and Gm21860, respectively, and 4931431B13Rik was not identified by the STRING.

Next, we examined whether the Control group shared some DEGs with the BPA group (Figs. 5, S4–S6, and Tables S3–S5). The results indicated that there were 16 genes in the intersection, all of which except *Cerkl* showed opposite expression trends (Table S6) in the Control and BPA groups. Furthermore, GO enrichment analysis found that DEGs in the intersection were mainly enriched in the *positive regulation of organization proteins*, which might participate in the regulation of the *peptide localization secretion extracellular* process enriched in the BPA group. Thus, genes in the intersections could participate in protein secretion. As a result, we distinguished 17 insulin secretion-related DEGs (*Stxbp5l*, *Fam3d*, *Mia3*, *Igf1*, *Hif1a*, *Aqp1*, *Kif5b*, *Tiam1*, *Map4k4*, *Cyp51*, *Pde1c*, *Rab3c*, *Arntl*, *Clock*, *Edn3*, *Kcnb1*, *and Krt20*) in the BPA group, and 15 regulator DEGs (*Zfp830*, *4931431B13Rik*, *Egr1*, *Ddit4l*, *Cep55*, *G530011O06Rik*, *Hspa1b*, *Hspa1a*, *Cox6a2*, *Ibtk*, *Banf1*, *Slc35b2*, *Golt1b*, *Lrp8*, *and Pttg1*) with opposite expression trends and 1 regulator DEG *Cerkl* with similar expression trends in the Control and BPA groups (Table S6). After that, their interaction network was conducted (Figs. 6, S7, S8) and the interactive nodes of each gene were counted (Table S7).

We checked these DEGs with keywords (*endocrine disruptor* and *pancreas)* on the Pubmed website and found that only *Igf1* was screened out. IGF-1 is a peptide hormone mainly produced by the liver and a major growth factor in adults^38^. It is structurally similar to insulin. Growth hormone stimulates the production of IGF-1. Wan et al. found that ex-vivo PFOS exposure suppressed the expression levels of IGF-1 receptor-β and insulin receptors in islets^39^. Monocrotophos (MCP) could also increase the levels of circulating IGF-1^40^. Similarly, maternal BPA exposure increased the *Igf2* expression in islets in whole F1 embryos, and male F1 and F2 offspring, which was related to altered DNA methylation^41^. However, the other DEGs still need to be further explored.

### DEGs-related diseases and their expressions in tumor and normal populations

Genes with not less than 5 interactive nodes were up-loaded in the CTD database (9 DEGs), and the top 5 diseases related to each key and regulator DEG were summarized in Table S9. As indicated, these DEGs were related to one or more cancer symptoms, such as weight loss, carcinoma, neoplasms, inflammation, hyperplasia, metastasis, and fibrosis diseases.

**Figure 7:**
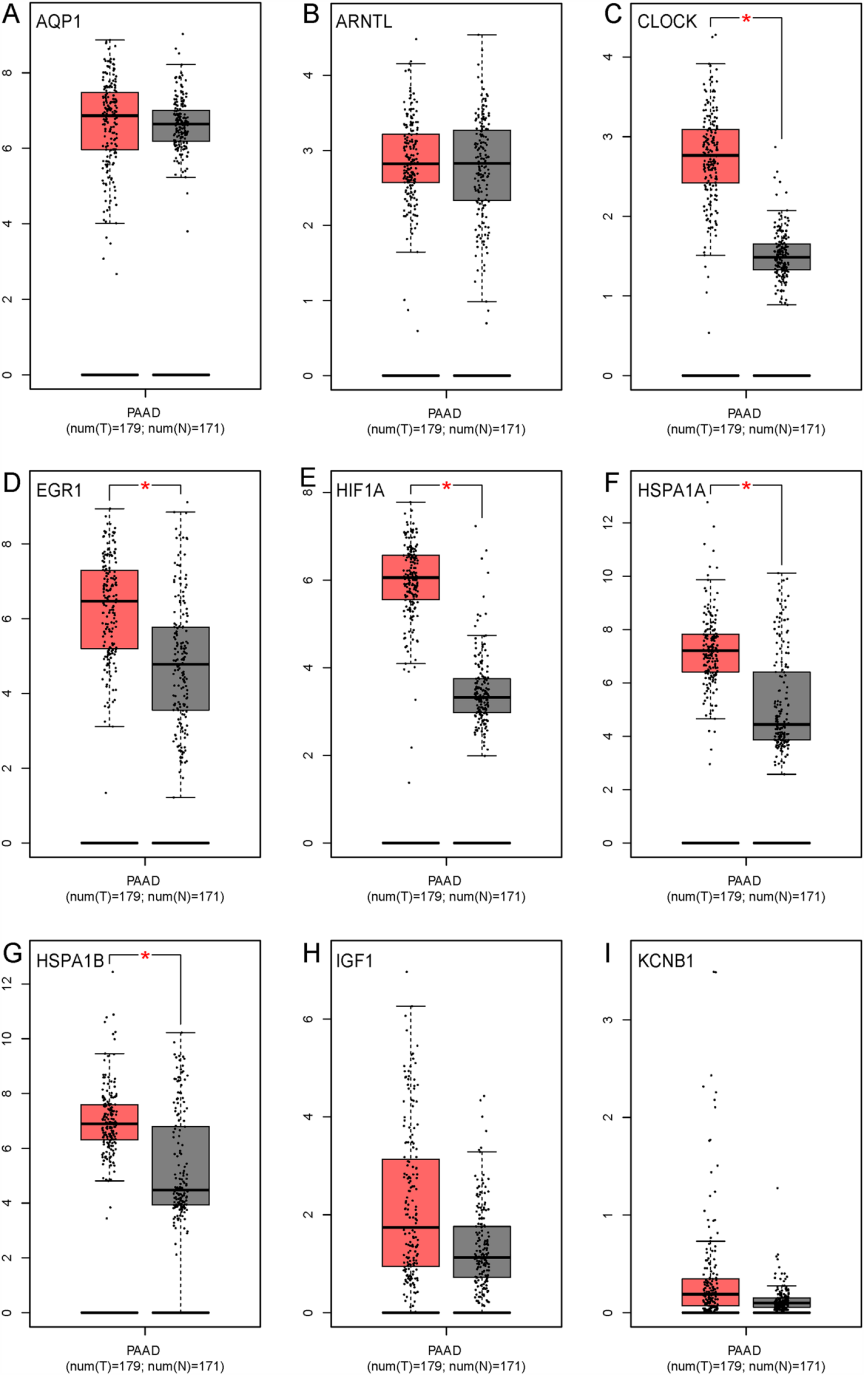
The expressions of the most interactive genes in tumor and normal populations. Red and grey color represents data in the tumor and normal population, respectively. *p < 0.05 represents a significant difference found between tumor and normal populations.

We further examined gene expressions in tumor and normal populations. As shown in Fig. 7, CLOCK, EGR1, HIF1A, HSPA1A, and HSPA1B were significantly up-regulated in tumor populations compared to normal populations, while the others remained unchanged. Considering that the expression of *Arntl*, *Clock*, *Hif1α* and *Kcnb1* was also up-regulated in the BPA group when comparing their expressions in offspring with those in adults (Table S8). The roles of *Clock* and *Hif1α* in the present study should be further investigated. As indicated, *Clock* plays a central role in the regulation of circadian rhythms and is associated with behavioral changes, obesity, and metabolic syndrome. *Hif1α* regulates the cellular and developmental response to reduced oxygen tension and plays a role in the induction of genes involved in cell proliferation and survival, energy metabolism, apoptosis, and glucose and iron metabolism (information collected from NCBI). To date, no researchers explored the important roles of *Clock* and *Hif1α* in direct or maternal BPA-induced pancreas diseases. Limited studies indicated that BPA exposure disturbed the expressions of circadian clock gene levels in mouse testes^42^, neurons of the hypothalamus^43^, mangrove killifish *Kryptolebias marmoratus*^44^, and female puberty and ovulation^32^. For *Hif1α*, BPA exposure could alter its expression in the heart of zebrafish^45,46^, placenta^47^, and brain^48^. In GSE82175, they also screened some cancer-related genes in the low-level BPA group, such as *Prss3* and *Agr2*.

**Figure 8:**
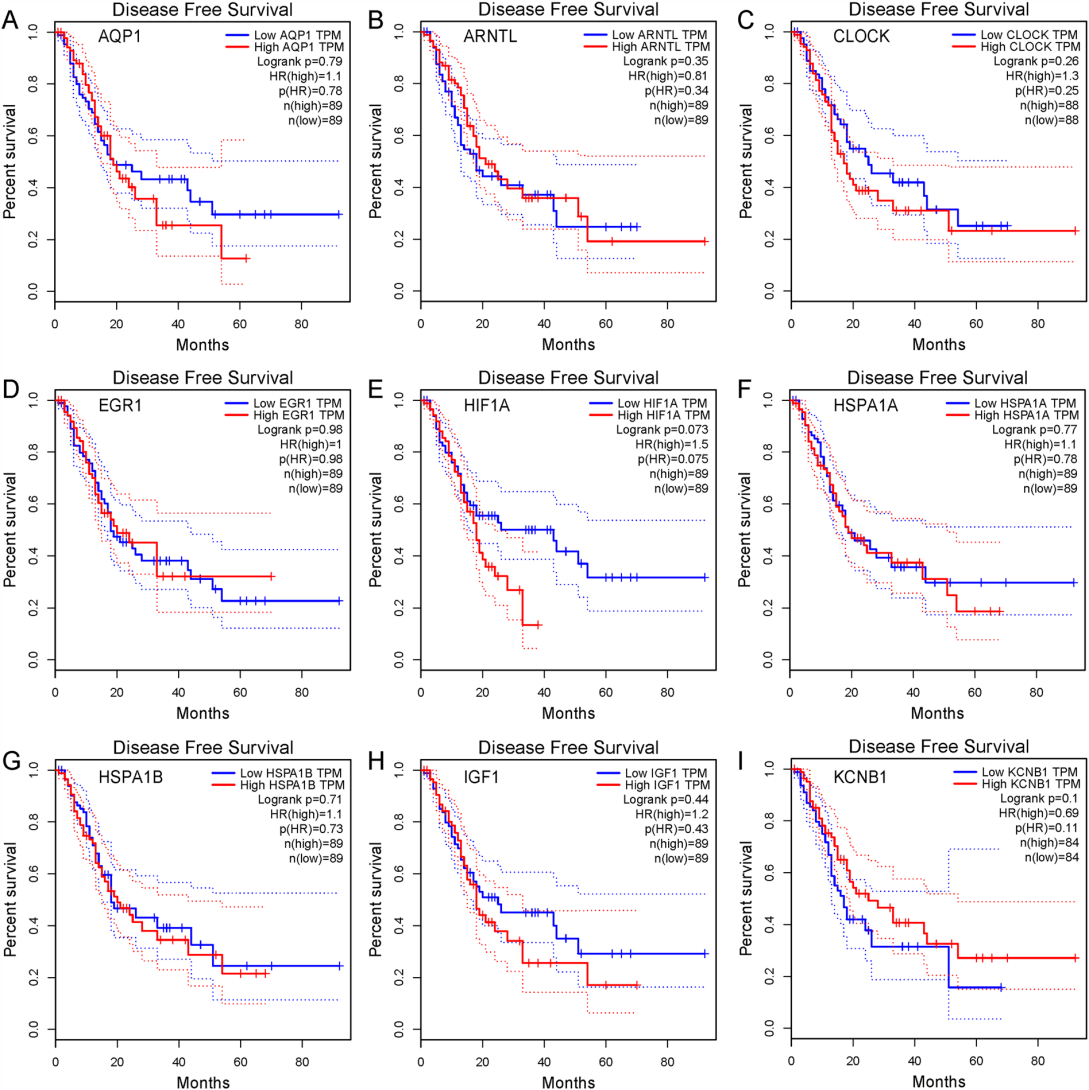
The disease-free survival curves of the most interactive genes with high and low TPM values. Red and blue lines represent data in high- and low-expressed samples collected at different time points.

Importantly, we explored the disease-free survival curve for each DEG (Fig. 8). The results showed that of HIF1A^high^ decreased the survival expectation (*p* = 0.073 for Logrank, HR (high) = 1.5). BPA has long been confirmed to be related to various cancers. Rodent studies^49,50^ and non-human primate studies^51^ indicated that prenatal exposure to BPA may disrupt the mammary tissue and increase the propensity to develop mammary cancer. Ho et al.^52^ reported that low-dose neonatal BPA exposure is associated with prostate cancer. Recent studies show that *Hif1α* might increase the risk of some cancers caused by BPA exposure^53,54^, and it might even participate in pancreatic cancer^55–57^. As a result, we speculated that *Hif1α* could be an important biomarker for BPA-enhanced pancreatic cancer risk.

## Conclusion

In this study, we found that pregnancy was a critical window of susceptibility to BPA exposure. *Hif1α* might be an important biomarker for BPA-enhanced pancreatic cancer risk. Our findings provide a theoretical basis for the diagnosis of pancreatic cancer induced by BPA.

## Supplementary Information

Supplementary Information.

## Data Availability

All data used in the present study were collected from the GEO database (GSE82175 and GSE126297). All data generated or analyzed during this study are included in this published article [and its supplementary information files].
