# Supplementary material for: Important roles of Hif1a in maternal or adult BPA exposure induced pancreatic injuries
Source: Sci Rep. 2023 Jul 17;13:11502. doi: 10.1038/s41598-023-38614-8 (PMC10352259; doi:10.1038/s41598-023-38614-8)
Supplement: Supplementary file 1 — Supplementary Information. [file 41598_2023_38614_MOESM1_ESM.pdf]

Important Roles of *Hif1α* in Maternal or Adult BPA Exposure Induced Pancreatic Injuries

Huiping Liu, Yongnian Zhou, Yike Li, Zhihua Gong*

Table S1. Top 30 GO terms predicted by all DEGs in the Control group.

Table S2. Top 30 GO terms predicted by all DEGs in the BPA group.

Table S3. Top 30 GO terms predicted by DEGs only in the Control group.

Table S4. Top 30 GO terms predicted by DEGs in both Control and BPA groups.

Table S5. Top 30 GO terms predicted by DEGs only in the BPA group.

Table S6. The list of 15 genes with opposite expression trends and 1 with similar expression trends in the Control and BPA groups.

Table S7. Numbers of interacted genes of each nodes.

Table S8. Roles, FC in the Control and BPA groups of 9 screened DEGs.

Table S9. Diseases predicted by the 9 most important genes.

Figure S1. GSEA for (A) the Control and (B) BPA groups.

Figure S2. GO enrichment of DEGs in the Control group.

Figure S3. GO enrichment of DEGs in the BPA group.

Figure S4. GO enrichment of DEGs only in the Control group.

Figure S5. GO enrichment of DEGs in both Control and BPA groups.

Figure S6. GO enrichment of DEGs only in the BPA group.

Figure S7. Gene interaction networks with default cut off (0.400) generated by genes participated in peptide regulation insulin secretion-related pathways and the genes included in the intersection between the Control and BPA groups.

Figure S8. Gene interaction networks with high confidence cut off (0.700) generated by genes participated in peptide regulation insulin secretion-related pathways and the genes included in the intersection between the Control and BPA groups.

Table S1. Top 30 GO terms predicted by all DEGs in the Control group.

ID	Description	pvalue	p.adjust	qvalue
GO:0051225	spindle assembly	6.00E-06	0.004108	0.003652
GO:0007052	mitotic spindle organization	7.32E-06	0.004108	0.003652
GO:0007051	spindle organization	7.52E-06	0.004108	0.003652
GO:0140014	mitotic nuclear division	1.32E-05	0.005416	0.004814
GO:0048285	organelle fission	2.34E-05	0.006483	0.005763
GO:1902850	microtubule cytoskeleton organization involved in mitosis	2.37E-05	0.006483	0.005763
GO:0000070	mitotic sister chromatid segregation	3.33E-05	0.007801	0.006934
GO:0007059	chromosome segregation	4.85E-05	0.009855	0.00876
GO:0000280	nuclear division	5.41E-05	0.009855	0.00876
GO:0000819	sister chromatid segregation	8.96E-05	0.014693	0.01306
GO:0090307	mitotic spindle assembly	0.000143	0.021299	0.018932
GO:0008608	attachment of spindle microtubules to kinetochore	0.000296	0.040462	0.035965
GO:0051983	regulation of chromosome segregation	0.000342	0.043114	0.038322
GO:0060236	regulation of mitotic spindle organization	0.000384	0.045	0.039999
GO:0045786	negative regulation of cell cycle	0.000416	0.045499	0.040442
GO:0034620	cellular response to unfolded protein	0.000464	0.047515	0.042234
GO:0090224	regulation of spindle organization	0.000526	0.04971	0.044185
GO:0051304	chromosome separation	0.000546	0.04971	0.044185
GO:0098813	nuclear chromosome segregation	0.000662	0.057092	0.050747
GO:0035967	cellular response to topologically incorrect protein	0.001046	0.079484	0.07065
GO:0051282	regulation of sequestering of calcium ion	0.001081	0.079484	0.07065
GO:0051208	sequestering of calcium ion	0.001153	0.079484	0.07065
GO:0009148	pyrimidine nucleoside triphosphate biosynthetic process	0.001164	0.079484	0.07065
GO:0051988	regulation of attachment of spindle microtubules to kinetochore	0.001164	0.079484	0.07065
GO:0006986	response to unfolded protein	0.001267	0.082217	0.073079
GO:0010457	centriole-centriole cohesion	0.001354	0.082217	0.073079
GO:0051315	attachment of mitotic spindle microtubules to kinetochore	0.001354	0.082217	0.073079
GO:1901216	positive regulation of neuron death	0.001565	0.091581	0.081403
GO:0010965	regulation of mitotic sister chromatid separation	0.001721	0.097253	0.086445
GO:0051306	mitotic sister chromatid separation	0.00198	0.10288	0.091446

Table S2. Top 30 GO terms predicted by all DEGs in the BPA group.

ID	Description	pvalue	p.adjust	qvalue
GO:0060537	muscle tissue development	5.61E-06	0.011424	0.00961
GO:0030593	neutrophil chemotaxis	7.36E-06	0.011424	0.00961
GO:0014706	striated muscle tissue development	9.59E-06	0.011424	0.00961
GO:0051146	striated muscle cell differentiation	2.53E-05	0.018037	0.015172
GO:0042692	muscle cell differentiation	3.27E-05	0.018037	0.015172
GO:0071621	granulocyte chemotaxis	4.36E-05	0.018037	0.015172
GO:1990266	neutrophil migration	4.36E-05	0.018037	0.015172
GO:0001655	urogenital system development	4.50E-05	0.018037	0.015172
GO:0099640	axo-dendritic protein transport	6.01E-05	0.018037	0.015172
GO:0008380	RNA splicing	6.90E-05	0.018037	0.015172
GO:0007519	skeletal muscle tissue development	7.08E-05	0.018037	0.015172
GO:0071902	positive regulation of protein serine/threonine kinase activity	7.27E-05	0.018037	0.015172
GO:0045860	positive regulation of protein kinase activity	7.60E-05	0.018037	0.015172
GO:0002790	peptide secretion	8.04E-05	0.018037	0.015172
GO:0042060	wound healing	8.18E-05	0.018037	0.015172
GO:0071347	cellular response to interleukin-1	8.68E-05	0.018037	0.015172
GO:0001822	kidney development	9.52E-05	0.018037	0.015172
GO:0014896	muscle hypertrophy	9.58E-05	0.018037	0.015172
GO:1901673	regulation of mitotic spindle assembly	9.59E-05	0.018037	0.015172
GO:0055001	muscle cell development	0.000109	0.019403	0.016321
GO:0060538	skeletal muscle organ development	0.000114	0.019403	0.016321
GO:0072001	renal system development	0.000153	0.024195	0.020352
GO:0006397	mRNA processing	0.000156	0.024195	0.020352
GO:0035914	skeletal muscle cell differentiation	0.00017	0.024805	0.020865
GO:0050708	regulation of protein secretion	0.000174	0.024805	0.020865
GO:0060326	cell chemotaxis	0.000204	0.026072	0.02193
GO:0006953	acute-phase response	0.000214	0.026072	0.02193
GO:0097530	granulocyte migration	0.000214	0.026072	0.02193
GO:0044403	biological process involved in symbiotic interaction	0.000215	0.026072	0.02193
GO:0050796	regulation of insulin secretion	0.000224	0.026072	0.02193

Table S3. Top 30 GO terms predicted by DEGs only in the Control group.

ID	Description	pvalue	p.adjust	qvalue
GO:0000070	mitotic sister chromatid segregation	0.000157	0.126302	0.111641
GO:0008608	attachment of spindle microtubules to kinetochore	0.000175	0.126302	0.111641
GO:0007051	spindle organization	0.000333	0.126302	0.111641
GO:0000819	sister chromatid segregation	0.000359	0.126302	0.111641
GO:0051225	spindle assembly	0.000554	0.126302	0.111641
GO:0007052	mitotic spindle organization	0.000631	0.126302	0.111641
GO:0007059	chromosome segregation	0.000777	0.126302	0.111641
GO:0009148	pyrimidine nucleoside triphosphate biosynthetic process	0.000817	0.126302	0.111641
GO:0051988	regulation of attachment of spindle microtubules to kinetochore	0.000817	0.126302	0.111641
GO:0010457	centriole-centriole cohesion	0.000952	0.126302	0.111641
GO:0051315	attachment of mitotic spindle microtubules to kinetochore	0.000952	0.126302	0.111641
GO:0048285	organelle fission	0.001163	0.137538	0.121573
GO:1902850	microtubule cytoskeleton organization involved in mitosis	0.001358	0.137538	0.121573
GO:0009147	pyrimidine nucleoside triphosphate metabolic process	0.001413	0.137538	0.121573
GO:0031145	anaphase-promoting complex-dependent catabolic process	0.001413	0.137538	0.121573
GO:0098813	nuclear chromosome segregation	0.001901	0.166239	0.146943
GO:0009142	nucleoside triphosphate biosynthetic process	0.002115	0.166239	0.146943
GO:0140014	mitotic nuclear division	0.002162	0.166239	0.146943
GO:0046697	decidualization	0.002163	0.166239	0.146943
GO:0006221	pyrimidine nucleotide biosynthetic process	0.002375	0.17335	0.153228
GO:0051983	regulation of chromosome segregation	0.00271	0.188433	0.16656
GO:0072528	pyrimidine-containing compound biosynthetic process	0.003064	0.197064	0.174189
GO:0000280	nuclear division	0.003104	0.197064	0.174189
GO:0045786	negative regulation of cell cycle	0.003787	0.224057	0.198049
GO:0051304	chromosome separation	0.003837	0.224057	0.198049
GO:0002724	regulation of T cell cytokine production	0.004396	0.246861	0.218206
GO:0006364	rRNA processing	0.004899	0.260262	0.230051
GO:0001893	maternal placenta development	0.004991	0.260262	0.230051
GO:0007007	inner mitochondrial membrane organization	0.005302	0.266931	0.235946
GO:0009141	nucleoside triphosphate metabolic process	0.005489	0.267112	0.236106

Table S4. Top 30 GO terms predicted by DEGs in both Control and BPA groups.

ID	Description	pvalue	p.adjust	qvalue
GO:0042026	protein refolding	5.86E-05	0.013543	0.008264
GO:1901673	regulation of mitotic spindle assembly	6.54E-05	0.013543	0.008264
GO:0090169	regulation of spindle assembly	0.000124	0.01497	0.009135
GO:0051085	chaperone cofactor-dependent protein refolding	0.000189	0.01497	0.009135
GO:0060236	regulation of mitotic spindle organization	0.00024	0.01497	0.009135
GO:0006458	'de novo' protein folding	0.000253	0.01497	0.009135
GO:0051084	'de novo' post-translational protein folding	0.000253	0.01497	0.009135
GO:0090224	regulation of spindle organization	0.000296	0.015324	0.009351
GO:0061077	chaperone-mediated protein folding	0.000581	0.025944	0.015831
GO:0140014	mitotic nuclear division	0.000662	0.025944	0.015831
GO:0042698	ovulation cycle	0.000689	0.025944	0.015831
GO:0090307	mitotic spindle assembly	0.000831	0.028669	0.017494
GO:0032436	positive regulation of proteasomal ubiquitin-dependent protein catabolic process	0.001428	0.04489	0.027393
GO:0034620	cellular response to unfolded protein	0.001525	0.04489	0.027393
GO:0009408	response to heat	0.001626	0.04489	0.027393
GO:2000060	positive regulation of ubiquitin-dependent protein catabolic process	0.001911	0.048709	0.029723
GO:1901800	positive regulation of proteasomal protein catabolic process	0.0021	0.048709	0.029723
GO:0035967	cellular response to topologically incorrect protein	0.002338	0.048709	0.029723
GO:0051225	spindle assembly	0.002379	0.048709	0.029723
GO:0000280	nuclear division	0.002455	0.048709	0.029723
GO:0007052	mitotic spindle organization	0.002546	0.048709	0.029723
GO:0006986	response to unfolded protein	0.002588	0.048709	0.029723
GO:1903052	positive regulation of proteolysis involved in cellular protein catabolic process	0.002762	0.049716	0.030338
GO:0032434	regulation of proteasomal ubiquitin-dependent protein catabolic process	0.003219	0.055535	0.033889
GO:0048285	organelle fission	0.003461	0.056363	0.034394
GO:0035966	response to topologically incorrect protein	0.00371	0.056363	0.034394
GO:1903364	positive regulation of cellular protein catabolic process	0.003761	0.056363	0.034394
GO:1902850	microtubule cytoskeleton organization involved in mitosis	0.003812	0.056363	0.034394
GO:0070507	regulation of microtubule cytoskeleton organization	0.004233	0.060433	0.036877

GO:0006457	protein folding	0.004507	0.061218	0.037356

Table S5. Top 30 GO terms predicted by DEGs only in the BPA group.

ID	Description	pvalue	p.adjust	qvalue
GO:0030593	neutrophil chemotaxis	4.91E-06	0.012068	0.010211
GO:0060537	muscle tissue development	9.72E-06	0.012068	0.010211
GO:0051146	striated muscle cell differentiation	1.41E-05	0.012068	0.010211
GO:0042692	muscle cell differentiation	1.72E-05	0.012068	0.010211
GO:0014706	striated muscle tissue development	1.73E-05	0.012068	0.010211
GO:0071621	granulocyte chemotaxis	2.95E-05	0.014737	0.01247
GO:1990266	neutrophil migration	2.95E-05	0.014737	0.01247
GO:0002790	peptide secretion	4.32E-05	0.015718	0.0133
GO:0071902	positive regulation of protein serine/threonine kinase activity	4.36E-05	0.015718	0.0133
GO:0042060	wound healing	4.64E-05	0.015718	0.0133
GO:0099640	axo-dendritic protein transport	4.94E-05	0.015718	0.0133
GO:0014896	muscle hypertrophy	6.75E-05	0.018833	0.015935
GO:0055001	muscle cell development	7.00E-05	0.018833	0.015935
GO:0001655	urogenital system development	9.08E-05	0.022676	0.019187
GO:0050708	regulation of protein secretion	0.000106	0.02461	0.020823
GO:0060326	cell chemotaxis	0.000124	0.024894	0.021064
GO:0009306	protein secretion	0.000132	0.024894	0.021064
GO:0008380	RNA splicing	0.000135	0.024894	0.021064
GO:0035592	establishment of protein localization to extracellular region	0.000135	0.024894	0.021064
GO:0097530	granulocyte migration	0.000147	0.024894	0.021064
GO:0050796	regulation of insulin secretion	0.000149	0.024894	0.021064
GO:0071692	protein localization to extracellular region	0.000164	0.025728	0.02177
GO:0006953	acute-phase response	0.000169	0.025728	0.02177
GO:0001822	kidney development	0.000212	0.029216	0.024721
GO:0010216	maintenance of DNA methylation	0.000215	0.029216	0.024721
GO:0007519	skeletal muscle tissue development	0.000217	0.029216	0.024721
GO:0006397	mRNA processing	0.000271	0.035068	0.029672
GO:0030072	peptide hormone secretion	0.000295	0.035068	0.029672
GO:0000380	alternative mRNA splicing, via spliceosome	0.0003	0.035068	0.029672
GO:0003300	cardiac muscle hypertrophy	0.00031	0.035068	0.029672

Table S6. The list of 15 genes with opposite expression trends and 1 with similar expression trends in the Control and BPA groups.

symbol	Con_FC	BPA_FC
Zfp830	0.43997	1.899398
Cerkl*	2.003221	1.568502
4931431B13Rik	0.411065	2.320241
Egr1	2.11475	0.393199
Ddit4l	0.217202	7.278313
Cep55	0.385366	1.526546
G530011O06Rik	0.484817	1.51423
Hspa1b	1.860885	0.528372
Hspa1a	1.802934	0.452452
Cox6a2	0.441415	2.047289
Ibtk	0.490362	1.790622
Banf1	1.985825	0.496204
Slc35b2	0.407312	2.404999
Golt1b	0.276245	2.544363
Lrp8	0.437159	2.2174
Pttg1	0.359077	1.68011

* with similar expression trends

Table S7. Numbers of interacted genes of each nodes.

Nodes	No. of interacted genes
Aqp1	6
Arntl	5
Banf1	2
Cep55	1
Clock	6
Cox6a2	1
Cyp51	3
Ddit4l	2
Edn3	4
Egr1	9
Golt1b	1
Hif1a	10
Hspa1a	6
Hspa1b	10
Ibtk	2
Igf1	10
Kcnb1	5
Kif5b	4
Lrp8	1
Map4k4	2
Pde1c	1
Pttg1	3
Rab3c	3
Slc35b2	2
Stxbp5l	2
Tiam1	1
Krt20	0
Cerkl	0
Fam3d	0
Mia3	0
4931431B13Rik	0
G530011O06Rik	0
Zfp830	0

Table S8. Roles, FC in the Control and BPA groups of 9 screened DEGs.

Gene	Role	FC BPA	FC Con
Aqp1	Core	0.46644	1.464996
Arntl	Core	1.51585	0.589349
Clock	Core	1.6205	0.791474
Egr1	Regulate	0.393199	2.11475
Hif1a	Core	1.512454	0.664951
Hspa1a	Regulate	0.452452	1.802934
Hspa1b	Regulate	0.528372	1.860885
Igf1	Core	0.457543	1.730592
Kcnb1	Core	1.527346	0.79522

Table S9. Diseases predicted by the 9 most important genes.

Genes	Disease Name	Disease ID	Direct Evidence	Inference Score	Cancer related
Aqp1	Hypertension	MESH:D006973	Marker or mechanism	131.69	-
	Carcinoma	MESH:D002277	Marker or mechanism	76.76	yes
	Pain	MESH:D010146	Marker or mechanism	70.64	-
	Mammary Neoplasms, Experimental	MESH:D008325	Marker or mechanism	64.44	yes
	Mammary Neoplasms, Animal	MESH:D015674	Marker or mechanism	61.41	yes
Arntl	Abnormalities, Multiple	MESH:D000015	Marker or mechanism	68.82	-
	Infertility, Female	MESH:D007247	Marker or mechanism	64.74	-
	Myocardial Ischemia	MESH:D017202	Marker or mechanism	53.69	-
	Leukemia, Myelogenous, Chronic, BCR-ABL Positive	MESH:D015464	Marker or mechanism	18.68	yes
	Hyperargininemia	MESH:D020162	Marker or mechanism	-	-
Clock	Weight Loss	MESH:D015431	-	189.62	yes
	Chemical and Drug Induced Liver Injury	MESH:D056486	-	176.79	-
	Hepatomegaly	MESH:D006529	-	170.18	-
	Inflammation	MESH:D007249	-	156.64	-
	Necrosis	MESH:D009336	-	156.29	-
Egr1	Inflammation	MESH:D007249	Marker or mechanism	448.65	yes
	Lung Neoplasms	MESH:D008175	Marker or mechanism	260.17	-
	Carcinoma, Hepatocellular	MESH:D006528	Marker or mechanism	155.39	yes
	Cholestasis	MESH:D002779	Marker or mechanism	106.53	-
	Prostatic Neoplasms	MESH:D011471	Marker or mechanism	95.37	yes
Hif1a	Hyperplasia	MESH:D006965	Marker or mechanism	272.21	yes
	Neoplasms, Experimental	MESH:D009374	Marker or mechanism	251.63	yes

	Cell Transformation, Neoplastic	MESH:D002471	Marker or mechanism	229.65	yes
	Breast Neoplasms	MESH:D001943	Marker or mechanism	201.11	yes
	Neoplasm Invasiveness	MESH:D009361	Marker or mechanism	188.21	yes
Hspa1a	Acute Kidney Injury	MESH:D058186	Marker or mechanism	184.77	-
	Brain Injuries	MESH:D001930	Marker or mechanism	145.35	-
	Liver Diseases	MESH:D008107	Marker or mechanism	136.57	-
	Neoplasm Metastasis	MESH:D009362	Marker or mechanism	113.03	yes
	Drug Hypersensitivity	MESH:D004342	Marker or mechanism	78.19	-
Hspa1b	Atherosclerosis	MESH:D050197	Marker or mechanism	91.84	-
	Skin Diseases	MESH:D012871	Marker or mechanism	59.98	-
	Arsenic Poisoning	MESH:D020261	Marker or mechanism	24.53	-
	Carcinoma, Pancreatic Ductal	MESH:D021441	Marker or mechanism	11.96	yes
	Muscular Dystrophy, Facioscapulohumeral	MESH:D020391	Marker or mechanism	-	-
Igf1	Chemical and Drug Induced Liver Injury	MESH:D056486	Marker or mechanism	591.61	-
	Cardiomegaly	MESH:D006332	Marker or mechanism	198.64	-
	Breast Neoplasms	MESH:D001943	Marker or mechanism	183.76	-
	Carcinoma, Hepatocellular	MESH:D006528	Marker or mechanism	154.78	yes
	Pulmonary Fibrosis	MESH:D011658	Marker or mechanism	141.73	yes
Kcnb1	Hernia, Diaphragmatic	MESH:D006548	Marker or mechanism	10.17	-
	Schizophrenia	MESH:D012559	Marker or mechanism	7.79	-
	DEVELOPMENTAL AND EPILEPTIC ENCEPHALOPATHY 26	OMIM:616056	Marker or mechanism	-	-
	Necrosis	MESH:D009336	-	181.83	-
	Weight Loss	MESH:D015431	-	171.87	yes

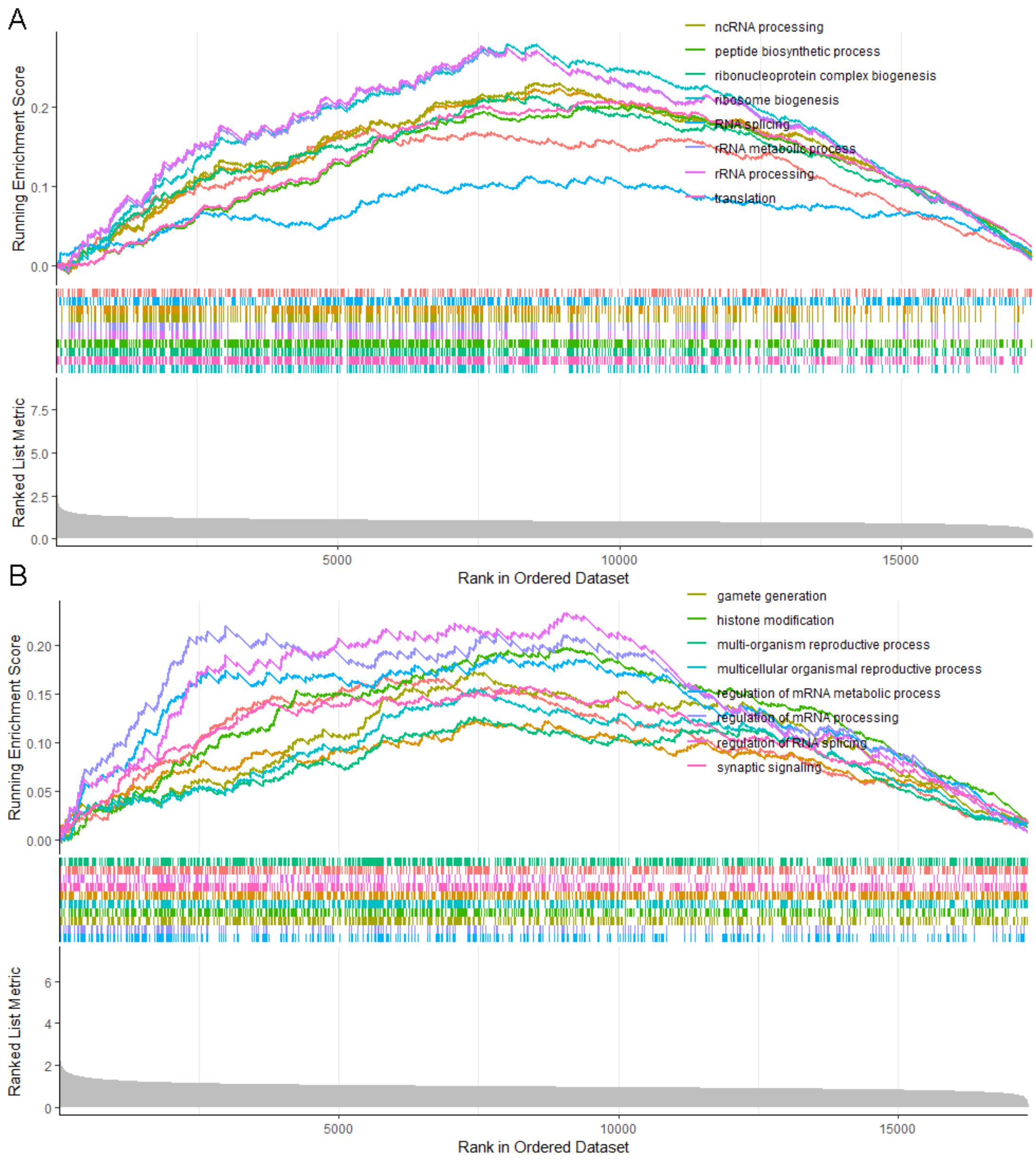

Figure S1. GSEA for (A) the Control and (B) BPA groups.

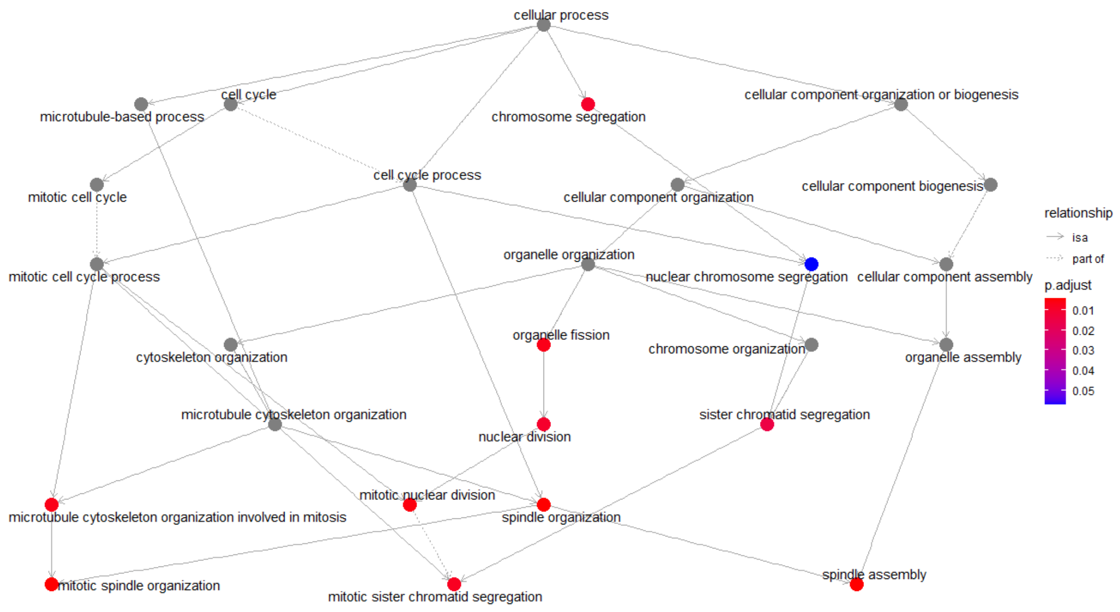

Figure S2. GO enrichment of DEGs in the Control group.

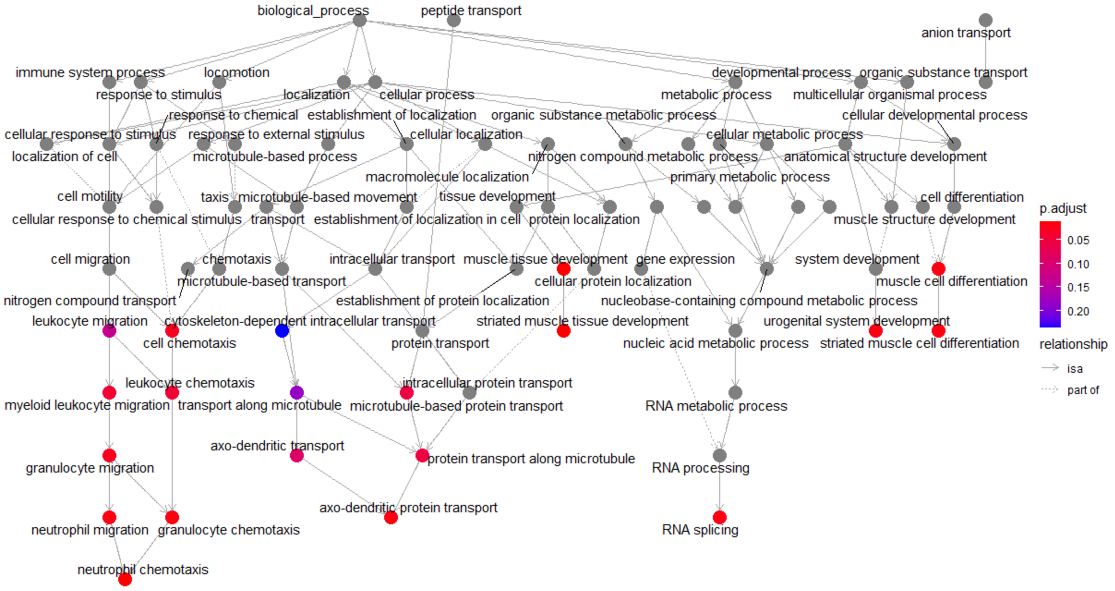

Figure S3. GO enrichment of DEGs in the BPA group.

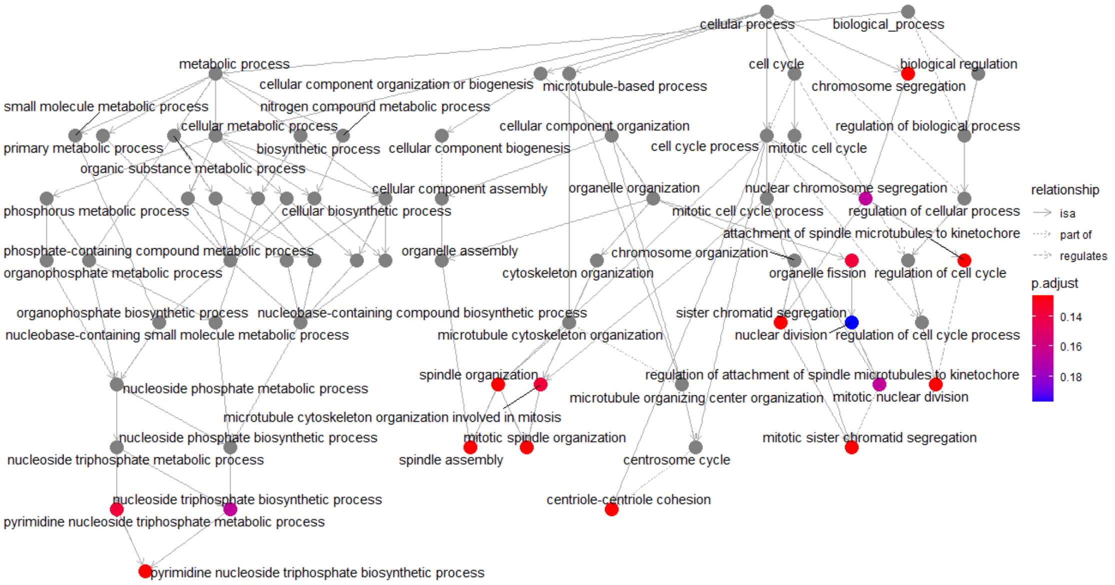

Figure S4. GO enrichment of DEGs only in the Control group.

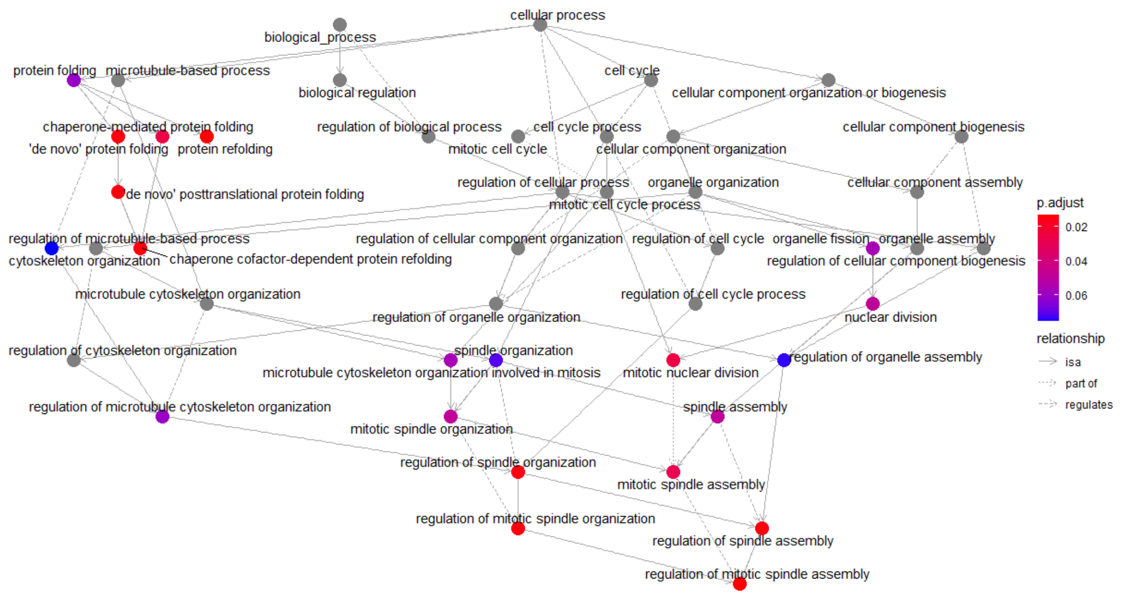

Figure S5. GO enrichment of DEGs in both Control and BPA groups.

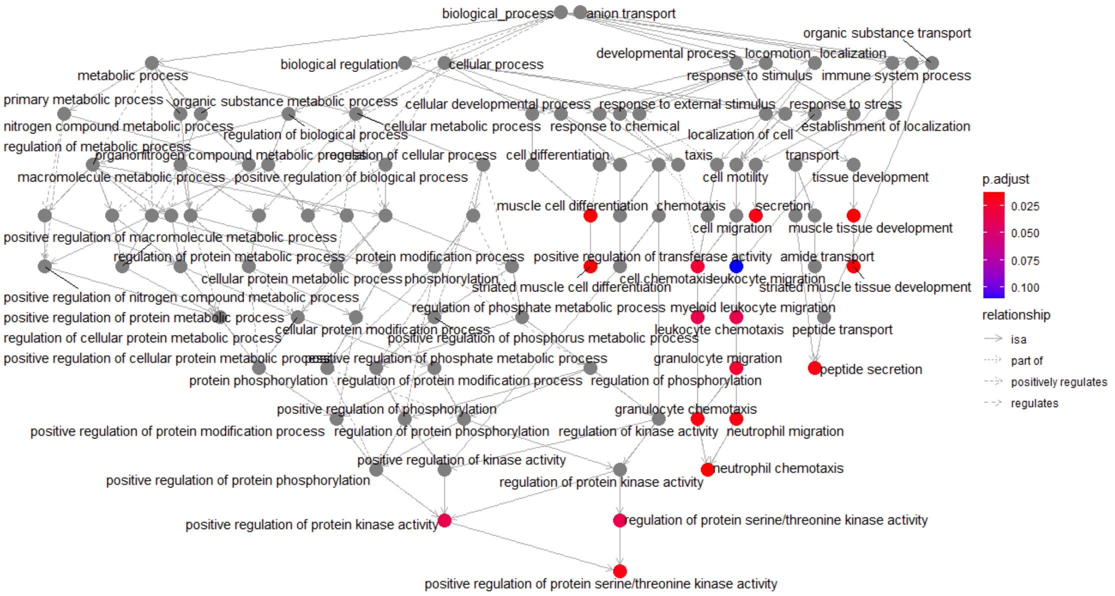

Figure S6. GO enrichment of DEGs only in the BPA group.

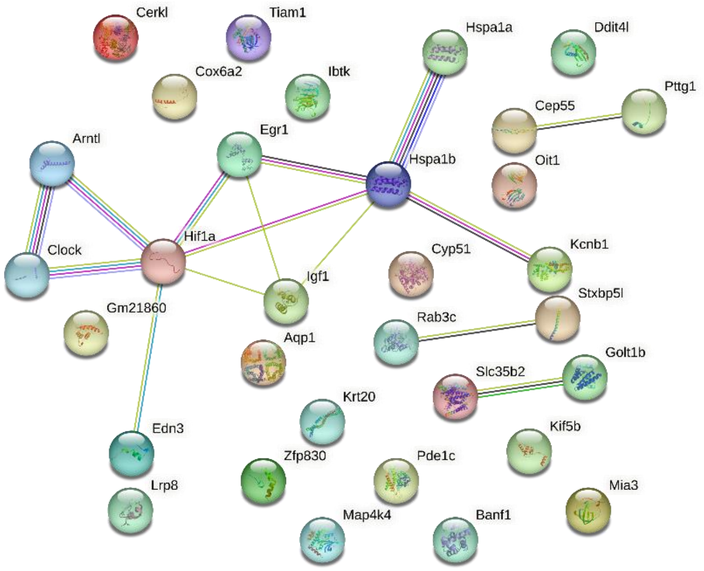

Figure S7. Gene interaction networks with default cut off (0.400) generated by genes participated in peptide regulation insulin secretion-related pathways and the genes included in the intersection between the Control and BPA groups. Fam3d and G530011O06Rik were recognized as Oit1 and Gm21860, respectively, and 4931431B13Rik was not identified by the STRING.

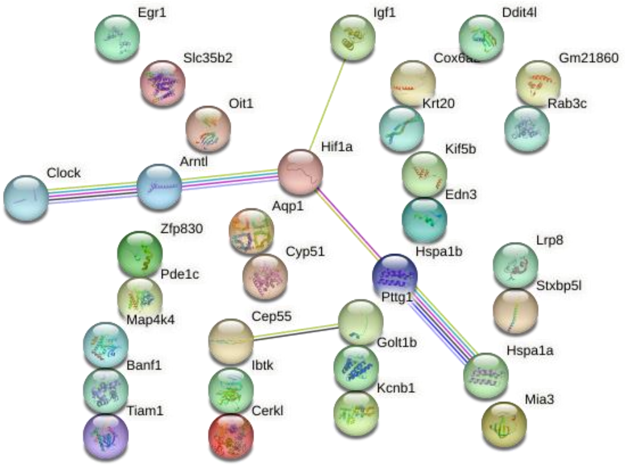

Figure S8. Gene interaction networks with high confidence cut off (0.700) generated by genes participated in peptide regulation insulin secretion-related pathways and the genes included in the intersection between the Control and BPA groups. Fam3d and G530011O06Rik were recognized as Oit1 and Gm21860, respectively, and 4931431B13Rik was not identified by the STRING.
